# Examining early inhibitory control and emotion regulation as predictors of childhood internalizing and externalizing problems: A longitudinal study

**DOI:** 10.1002/jcv2.70093

**Published:** 2026-01-07

**Authors:** Lilja K. Jónsdóttir, Matilda A. Frick, Andreas Frick, Emma J. Heeman, Karin C. Brocki

**Affiliations:** ^1^ Department of Psychology Uppsala University Uppsala Sweden; ^2^ Centre for Women's Mental Health during the Reproductive Lifespan – WOMHER Uppsala University Uppsala Sweden; ^3^ Department of Psychology Stockholm University Stockholm Sweden; ^4^ Department of Medical Sciences, Child and Adolescent Psychiatry Uppsala University Uppsala Sweden; ^5^ Department of Medical Sciences, Experimental Cognitive and Affective Neuroscience Lab (ECAN Lab) Uppsala University Uppsala Sweden

**Keywords:** emotion regulation, externalizing problems, inhibitory control, internalizing problems, self‐regulation

## Abstract

**Background:**

Identifying predictors and mechanisms in the development of childhood internalizing (INT) and externalizing (EXT) problems is crucial for early intervention. Inhibitory control has been linked to INT and EXT, with emotion regulation (ER) potentially mediating these associations. However, specific pathways between early inhibitory control, ER, and later INT and EXT remain unclear. Additionally, regulation of distinct emotions (anger, fear, sadness, joy) may play a role.

**Methods:**

The sample included 94 typically developing children from the EFFECT study, a longitudinal project on the development of self‐regulation. At age 4, inhibitory control was measured using the Day/Night Stroop Task. At age 6, general ER, as well as regulation of specific emotions (anger, fear, sadness, and joy), were assessed using the Emotion Questionnaire (parent‐report). INT and EXT at ages 9–10 were measured using the Strengths and Difficulties Questionnaire (parent‐report). Correlational and path analyses were conducted.

**Results:**

No longitudinal associations were found between inhibitory control at 4 years and either INT or EXT at ages 9–10, or with ER at age 6. Consequently, we found no evidence of mediation by ER. General ER at 6 years emerged as a predictor of both INT and EXT at 9–10 years. While not statistically significant, effect sizes linking regulation of some specific emotions (anger, fear) with subsequent INT and EXT problems warrant further research.

**Conclusion:**

The results reflect the complexity of studying longitudinal effects of early inhibitory control. A modest sample size with attrition, and measurement constraints may have attenuated effects and limited generalizability. Meanwhile, our findings highlight ER as a target for intervention across both INT and EXT.

## Key Points

**What's known?**

- Early inhibitory control is proposed to play a role in the development of behavioral and emotional problems, but specific longitudinal associations with internalizing (INT) and externalizing (EXT) problems are not known. Emotion regulation (ER) may play a mediating role, but this has not been previously studied.

**What's new?**

- We did not find evidence of a longitudinal predictive role of inhibitory control in the development of INT or EXT problems, and no evidence of a mediation effect.
- However, general ER in early childhood emerged as a longitudinal predictor of both INT and EXT problems.

**What's relevant?**

- General ER should be targeted for early intervention.
- Future studies should use richer measurement of inhibitory control, designs with shorter intervals between measures, and, larger, clinically enriched samples.

## Graphical abstract

In a longitudinal sample (*n* = 94), we tested links between inhibitory control at age 4, emotion regulation (ER) at age 6, and internalizing (INT) and externalizing (EXT) problems at ages 9–10. Early inhibitory control did not predict ER or later INT/EXT (no mediation), whereas ER at 6 showed prospective associations with both outcomes. Despite modest sample and single‐timepoint measures, findings highlight ER as a target for prevention across INT and EXT.

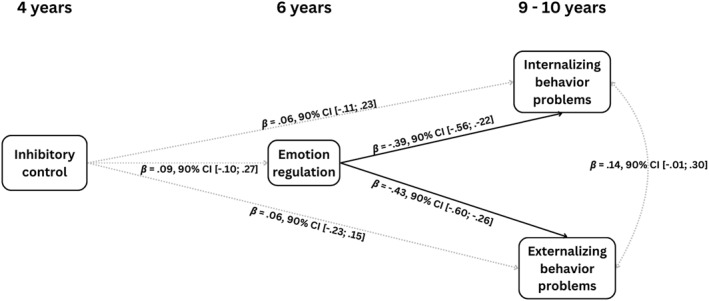

## INTRODUCTION

Behavioral, emotional, and social problems are common in childhood and, when persistent or severe, can impair daily functioning or indicate clinically relevant symptoms. These problems often co‐occur and are commonly summarized by two correlated yet distinct dimensions: internalizing (INT; inwardly directed problems such as worry, sadness, and social withdrawal) and externalizing (EXT; outwardly directed problems such as impulsivity, defiance, and aggression) (Achenbach et al., 2016; Cicchetti & Natsuaki, 2014). While INT and EXT represent dimensions of clinical symptoms, elevated levels in non‐clinical community samples are also robustly associated with concurrent and later maladjustment and psychopathology (Fanti & Henrich, 2010; Kassing et al., 2019; Mason et al., 2004; Roza et al., 2003; Vergunst et al., 2023). Although aspects of both INT and EXT follow heterogeneous developmental courses, in broader terms EXT often emerge early in childhood and decrease over time (Côté et al., 2006; Fanti & Henrich, 2010; Gilliom & Shaw, 2004), whereas INT have a later onset through a more gradual increase (Beesdo et al., 2009; Hankin et al., 2015). By late childhood, EXT are therefore typically well‐established, while INT show increasing stability (Fanti & Henrich, 2010), which is consistent with the timing of onset of various behavioral and emotional disorders (Solmi et al., 2022). To facilitate development of successful early interventions, it is crucial to identify indicators of the emergence of INT and EXT in childhood, and to delineate possible underlying developmental mechanisms.

Executive function is an important childhood predictor of later INT and EXT (Bloemen et al., 2018; Halse et al., 2022; Lynch et al., 2021; Romer & Pizzagalli, 2021; Yang et al., 2022). A proposed mechanism in this association is the specific role of inhibitory control, the top‐down (effortful) ability to override a prepotent action or thought that is incompatible with a higher order cognitive goal (Anderson & Weaver, 2009; Hofmann et al., 2012; Nigg, 2017), in the regulation of attention, behavior, and emotion (Carver et al., 2017; Joormann, 2010; Nelson et al., 2019). Inhibitory control emerges early and can be detected already in infancy (Holmboe et al., 2018), and individual differences become moderately stable and reliably measurable by 2 years of age (Broomell & Bell, 2022). The period between 3 and 5 years is marked by especially rapid improvement (Carlson, 2005; Garon et al., 2008, 2014; Wiebe et al., 2012). While development continues throughout middle childhood and adolescence, early childhood represents a foundational window of time in which inhibitory control capacities are both highly plastic and predictive of future outcomes. At face value, this general ability is as essential for inhibiting rumination and negative thoughts (aspects of INT) as it is for inhibiting impulsive behavior or aggression (aspects of EXT), indicating a role for early inhibitory control in the development of both INT and EXT. However, this picture is complicated by indications of potential non‐linear or negative associations between inhibitory control and socioemotional outcomes (e.g., Carlson & Wang, 2007; Hendry et al., 2022; Hsieh & Stright, 2012). Previous studies have consistently demonstrated a cross‐sectional and longitudinal association between inhibitory control and EXT in childhood (Berger & Buttelmann, 2022; Bohlin et al., 2012; Gagne et al., 2019; Kahle et al., 2018). Meanwhile, research linking inhibitory control to INT is limited and inconsistent (Backer‐Grøndahl et al., 2019; Berger & Buttelmann, 2022; Blanken et al., 2017; Brocki & Bohlin, 2006; Eisenberg et al., 2005). Moreover, INT and EXT are often examined separately, precluding examination of specific associations (Achenbach et al., 2016). Considering high comorbidity between INT and EXT (Danielson et al., 2021; Lahey et al., 2017; Willner et al., 2016), and the relative scarcity of studies, there is need for more research on the longitudinal, specific associations between early inhibitory control and later INT and EXT.

A potential mediator in the pathway between inhibitory control in toddlerhood and later INT and EXT is the development of ER. While definitions vary, ER can be broadly described as the ability to successfully modulate the intensity, duration, and quality of an affective state, experience, or response, in the service of a higher order cognitive goal (Eisenberg et al., 2007; Gross, 2015). In infancy and early toddlerhood, successful ER depends heavily on caregiver‐supported processes, with a gradual shift toward greater reliance on internal regulation strategies across toddlerhood and early childhood (Cole et al., 2004; Eisenberg & Spinrad, 2004). Emotion regulation (ER) is actively improving during the transition into school‐age (Blandon et al., 2008; Murray et al., 2025; Noroña et al., 2018), as internal regulation strategies develop and become more sophisticated (Kopp, 1989). By around 6 years, these abilities are increasingly child‐directed but still highly plastic, providing a meaningful point at which earlier influences such as inhibitory control can shape emerging regulatory skills, even as ER continues to mature throughout later childhood and adolescence (e.g., Silvers, 2022). Inhibitory control has strong theoretical ties with ER (Bartholomew et al., 2021; Joormann, 2010, 2019; Ochsner et al., 2012; Pruessner et al., 2020), but empirical support for a longitudinal association in early childhood is limited (Carlson & Wang, 2007; Hughes et al., 2023). Theory and empirical evidence indicate that higher levels of inhibitory control lead to more successful ER (Bartholomew et al., 2021; Hughes et al., 2023; Pruessner et al., 2020; but for a non‐linear perspective, see Carlson & Wang, 2007; Hendry et al., 2022). In turn, ER has been described as a robust, transdiagnostic factor in childhood behavior and emotional problems, and psychopathology (Aldao et al., 2016; Beauchaine, 2015; Cavicchioli et al., 2023; Compas et al., 2017; Fernandez et al., 2016). While there is extensive cross‐sectional and longitudinal evidence of the role of ER in both INT and EXT (Cavicchioli et al., 2023; Compas et al., 2017), few longitudinal studies include INT and EXT concurrently as outcomes (see Cavicchioli et al., 2023). Our understanding of how ER in early childhood may differentially contribute to the development of later INT and EXT is therefore limited.

Furthermore, the role of ER in the aforementioned pathway between inhibitory control and INT and EXT may depend on the type or quality of emotion being regulated. Although ER is most often defined and examined as a global capacity, different emotions may require unique strategies (Endrerud & Vikan, 2007; Rivers et al., 2007; Zimmermann & Iwanski, 2014), suggesting distinct associations between early inhibitory control and later regulation of specific emotions. In turn, regulation of specific emotions like anger, fear, sadness, and joy may impact development of INT and EXT differently (Eisenberg et al., 2001). Though research on independent effects is limited, dysregulation of negative emotions is linked to both INT and EXT (Beauchaine, 2015; Finlay‐Jones et al., 2024). Regulation of positive emotions (from this point referred to as *joy*), to prevent maladaptive and/or inappropriate excitement, is less studied (Brocki et al., 2019; Rydell et al., 2003; Vogel et al., 2019, 2023). However, the limited evidence available indicates that such regulation of joy plays a role in EXT specifically (Brocki et al., 2019; Rydell et al., 2003; Vogel et al., 2019).

Taken together, previous research highlights inhibitory control as an important factor in the development of INT and EXT, and that ER is affected by inhibitory control and further affects INT and EXT. Further understanding of these associations may inform early interventions, potentially reducing risk of later childhood psychopathology and maladjustment. Given theoretical grounding coupled with a scarcity of empirical evidence, the aim of our study was threefold; (1) to examine the specific longitudinal associations between inhibitory control in toddlerhood (4 years), ER at 6 years, and INT and EXT, respectively, in late childhood (9–10 years), in a typically developing sample; (2) to examine whether the potential longitudinal associations between inhibitory control and INT and EXT are mediated by ER in early childhood (6 years); and (3) to examine the possibly distinct roles of regulation of specific emotions in these associations.

Based on prior empirical evidence and theory, we expect positive associations between inhibitory control and ER, both generally and for specific emotions. We expect that inhibitory control at age 4 and ER at age 6 will be negatively associated with INT and EXT at ages 9–10. We also hypothesize that ER at age 6 will partially mediate the associations between inhibitory control, and both INT and EXT. Specifically, we anticipate partial mediation effects of anger, sadness, and fear regulation on these associations. For joy, we expect a partial mediation effect with EXT, while the role of joy regulation in relation to INT remains an open question.

## METHODS

### Participants

Data were drawn from the EFFECT study; a longitudinal project on the development of self‐regulation (Frick et al., 2017, 2019; Heeman et al., 2024; Jónsdóttir et al., 2024). Data from the project's 5th, 6th, and 7th wave were analyzed (at ages 4, 6, and 9–10 years). The sample was recruited from the birth registry in Uppsala, Sweden. Families in the area with newborns received a letter informing the parents of the project and asking them to take part in the study. The response rate was 30% (*n* = 146). Initial exclusion criteria were atypical development, preterm birth (child was born before 37 weeks gestational age; *n* = 2), and parents not speaking Swedish with the child (*n* = 6). Exclusion and further attrition (see Appendix S1) resulted in an initial sample of 126 eligible infants and their parents at baseline. Of these, 94 children provided data at two or more of the three follow‐up timepoints (ages 4, 6, and 9–10 years), and were therefore included in the present analyses (49 boys, 52.1%; 45 girls, 47.9%). As not all children contributed data at every wave of data collection, the number of participants differed across timepoints: 87 of the participants provided data at T1, 92 of the participants at T2, and 79 of the participants at T3. The majority of the children's parents were born in Sweden (95.2% of mothers, 90.4% of fathers). 2.9% of both mothers and fathers were born elsewhere in Europe). 1.9% of mothers and 6.7% of fathers were born outside of Europe.

Two participants stopped participation in the project after the first timepoint. A qualitative comparison of socio‐economic status (SES) and baseline measure of inhibitory control revealed a lower SES score (*M* = 4.0) and a higher score on inhibitory control (*M* = 8.5) for these two participants than observed for included participants (see Table 1).

**TABLE 1:** Sample characteristics and descriptive statistics of study variables and covariates.

	*n*	*M* (SD)/%	Range	% Missing	Skewness (absolute *z*‐score)	Kurtosis (absolute *z*‐score)
T1 (4 years)						
Age in months	87	47.5 (0.6)	46–49	0		
Day/Night Stroop	74	6.4 (3.6)	0–12	21.3	−1.5	−1.8
T2 (6 years)						
Age in months	92	73.0 (3.8)	68–82	0		
Emotion regulation (general)	90	28.9 (5.8)	15–39	4.3	−2.1	−0.5
Reg. of anger	90	3.6 (0.9)	1–5	4.3		
Reg of sadness	90	3.7 (0.8)	1.5–5	4.3		
Reg of fear	90	3.7 (0.8)	1.5–5	4.3		
Reg of joy	90	3.6 (0.9)	1.5–5	4.3		
T3 (9–10 years)						
Age in months	79	112.7 (5.3)	105–122	0		
SDQ internalizing	79	12.9 (2.5)	10–21	16.0	3.4	1.2
SDQ externalizing	79	14.3 (4.3)	10–26	16.0	2.9	−0.6
Covariates						
SES	90	4.5 (0.7)	2.8–5.5	4.3	−2.3	−0.8
Sex	94			0		
Boys	49	52.1				
Girls	45	47.9				

The study was approved by the Regional Ethical Board in Uppsala and conducted in accordance with the 1964 Declaration of Helsinki. Written informed consent was provided by the children's legal guardians at each timepoint of the study. Participation was rewarded with gift cards (worth approximately 18 EUR) after each occasion of data collection.

### Procedure

At 4 years (timepoint 1; T1), inhibitory control was assessed during a visit to lab facilities at Uppsala University along with a guardian. At 6 years (timepoint 2; T2), ER was assessed using an online parent‐report questionnaire. At 9–10 years (timepoint 3; T3), emotional and behavioral problems (INT and EXT) were assessed using an online parent‐report questionnaire.

The task and questionnaires in this study were part of a larger battery of behavioral assessments and questionnaires assessing cognitive and emotional processes in infancy and childhood. The broader study battery varied across timepoints, in that not all constructs were assessed at every age. Data availability therefore differed across timepoints (data on inhibitory control was limited to age 4, ER to age 6, and INT/EXT to age 9–10). Background information (SES, child biological sex, age) was collected through questionnaires. Data on inhibitory control at 4 years, and its association with SES in infancy, and child biological sex, has been previously analyzed and published (Jónsdóttir et al., 2024). At this time, no additional associations presented in this study have been published elsewhere.

### Measurements

*Inhibitory control* was measured using a computerized version of the Day/Night Stroop task (Gerstadt et al., 1994). Previous research indicates informative variation in performance at this age (Montgomery & Koeltzow, 2010). Adequate test‐retest reliability has been found for this task (in a slightly different version) when used with children in the same age range as in the current study (Thorell & Wåhlstedt, 2006). Pictures of a sun and a moon were displayed on a computer screen, and the experimenter discussed with the child how the sun shines during the day, and the moon shines at night. After this, the child was invited to play an “opposite game”; say “night” when shown a picture of a sun, and “day” when shown a picture of a moon. Pictures were shown, twice each, for familiarization, and feedback was provided. Next, a test trial was conducted, with 12 pictures presented in a fixed order for 2.5 s each, with 2.5 s pauses between picture presentations. The child's first answer was recorded. Possible scores ranged from 0 to 12 correct answers.

*ER* was measured using the Swedish version of the shortened Emotion Questionnaire (EQ; Rydell et al., 2003), a parent report questionnaire. While the EQ includes a total of 16 items that measure both reactivity and regulation, items pertaining to regulation only (8 items) were selected as a measure of ER (as in e.g., Frick et al., 2022). Items assess the child's ability to regulate emotions (anger, fear, joy, sadness) independently or with support. Example items include “*It is easy for others, for instance a parent, to calm him/her down*,” and “*He/she has difficulties calming down on his/her own*” (reversed item). Parents rated items on a 5‐point scale (1 = *does not apply at all*, to 5 = *applies very well to my child*). Previous research has indicated reliability, construct and predictive validity, and internal consistency of the scale (Brocki et al., 2019; Frick et al., 2022; Rydell et al., 2003). Internal consistency (Cronbach's alpha) of the whole scale was high in the current sample; *α* = 0.89. Internal consistencies of measures of regulation of each emotion were somewhat lower, albeit acceptable with only two items, ranging from *α* = 0.60 (regulation of sadness) to *α* = 0.75 (regulation of anger).

*INT and EXT* were assessed using the second‐order INT and EXT subscales from the Swedish Strengths and Difficulties Questionnaire (SDQ; Goodman et al., 2010; Goodman, 1997), a parent‐report measure. The SDQ includes 25 items across five subscales (emotional problems, peer problems, behavioral problems, hyperactivity, and prosocial behavior). These represent broader INT (emotional, peer problems) and EXT (behavioral, hyperactivity) scales, plus a Prosocial scale (Caci et al., 2015), which was excluded as it doesn't measure emotional or behavioral problems. Research suggests that second‐order INT and EXT scales are more informative than the five built‐in subscales of the questionnaire for typically developing samples, as in this study (Goodman et al., 2010). Each broad subscale therefore consists of 10 items in the form of statements, with the parent or guardian rating items on a 3‐point scale (1 = *not true*, to 3 = *certainly true*). Example items from the INT subscale include; “*Often unhappy, depressed or tearful*,” and “*Picked on or bullied by other children*.” Example items from the EXT subscale include; “*Restless, overactive, cannot stay still for long*,” and “*Often fights with other children or bullies them*.” Convergent and discriminant validity between the INT and EXT subscales has been shown to be satisfactory (Goodman et al., 2010). Internal consistency differed between the two subscales in the current sample; *α* = 0.63 for the INT subscale, and *α* = 0.88 for the EXT subscale.

### Covariates

Child biological sex and SES were included as covariates in the study models. Previous research has shown sex differences in self‐regulation (Matthews et al., 2009; van Tetering et al., 2020; Weis et al., 2013) and ER (Nolen‐Hoeksema, 2012; Sanchis‐Sanchis et al., 2020). SES is a strong predictor of child psychopathology (Guhn et al., 2020) and may influence ER (Hao & Farah, 2020) and self‐regulation (Backer‐Grøndahl & Nærde, 2017).

To calculate SES, we used an index based on the combined average of parental education and parental income (from both parents, if applicable). Parental education was measured on a 5‐point scale (1 = *Elementary school (9 years or less)*, to 5 = *University education*). Parental income was measured on a 6‐point scale (1 = *0–100,000 SEK*, to 6 = *Above 500,000* SEK). The median yearly income in the area was about 272,000 SEK at the time.

### Statistical analysis

To answer our main research questions, we used correlational analyses of bivariate associations, and path analyses of theoretical models. Preliminary analyses were therefore conducted to verify assumptions underlying these analyses, and to maximize the statistical power and quality of our data. All analysis was conducted in R Studio (v. 2024.4.2.764; Posit Team, 2024), and the full analysis code is available in Appendix S2.

Given the theoretical nature of this research, parameter‐rich models, and the relatively small sample size, we opted to set the alpha level threshold at 0.10. This decision was made to facilitate detection of possible emerging relationships and patterns in the data. Descriptive statistics and histograms were produced for each of the continuous study variables. Variables with skewness and kurtosis within absolute *z* = ±3.29 were deemed to be approximately normally distributed, as recommended for medium‐sized samples (Kim, 2013). As INT scores on the SDQ were right‐skewed, and EXT scores approached the cutoff, both were Box‐Cox transformed to ensure uniformity; other variables were approximately normal (see Table 1).

All continuous study variables were screened for univariate outliers using Median Absolute Deviation analysis (MAD; Leys et al., 2019). Two low outliers on sadness regulation and one on fear regulation were Winsorized (i.e., extreme values replaced by the 5% quantile value). As sensitivity analyses (correlational and path analysis) indicated no effect on results, these were retained. Variables were also screened for multivariate outliers using the Mahalanobis distance. Six observations exceeding the critical value of 0.01 were flagged as potential multivariate outliers. A sensitivity analysis (analysis of path models excluding potential outliers from the dataset) revealed no difference in main findings. The potential outliers were therefore retained.

At T2, two participants provided data after the children had surpassed the age limit for participation (at 81–82 months). However, excluding their data did not affect findings, so their data was retained in the final analysis.

Several variables had missing values (see Table 1). No meaningful patterns of missingness were detected, suggesting potential missingness not at random. To maximize power, we performed multiple imputation through chained equations, using the *mice* package in R (v. 3.16.0; van Buuren & Groothuis‐Oudshoorn, 2011). A sensitivity analysis using listwise deletion revealed a discrepancy in results, indicating possible bias among participants with complete data and resulting in a reduced sample (*n* = 55). Therefore, listwise deletion results were not interpreted. To minimize bias, we included theoretically relevant auxiliary variables (see Appendix S3) in the imputation model. These were measured either concurrently or at a previous timepoint. Due to scale differences, all numeric variables were standardized prior to imputation (as recommended by Woods et al., 2021). All subsequent analysis was performed using 21 imputed datasets (number based on the highest percentage of missing data on a single variable), and results described below were derived by pooling results using the R package *lavaan.mi* (v. 0.1–0.0029; Jorgensen, 2024).

## RESULTS

### Sample characteristics and descriptive statistics

Table 1 presents descriptive statistics for all study variables. INT scores on the SDQ were right‐skewed, and EXT scores approached the cutoff. INT and EXT scores showed a large range, from no reported problems to values corresponding to the 95th percentile based on Swedish subscale norms for children of the same age (corresponding to 20 points on the current study's scale; Björnsdotter et al., 2013). The range of EXT scores was somewhat larger than that of INT, and a greater percentage of children scored above the clinical cutoff values on the EXT scale. Specifically, 6% of children scored above the 90th percentile on INT compared to 21% above the 90^th^ percentile on EXT. Due to skewness, INT and EXT were Box‐Cox transformed to ensure uniformity; other variables were approximately normal. While a small number of univariate and multivariate outliers were identified, sensitivity analyses showed they did not affect the main results; all cases were therefore retained. Missing data varied across measures but did not exceed 21%. Diagnostic plots indicated adequate correspondence between the observed and imputed data (see Supporting Information S1: Appendix S3).

### Bivariate associations

An analysis of bivariate correlations revealed that inhibitory control at 4 years was not associated with INT or EXT at 9–10 years, and no association was found between inhibitory control at 4 years and ER at 6 years (general or specific emotions; see Table 2).

**TABLE 2:** Bivariate associations between study variables and proposed covariates (Pearson zero‐order correlations and point biserial correlations), with 95% confidence intervals (*α* = 0.10).

	1	2	3	4	5	6	7	8	9	10
T1										
1. Inhibitory control	1.00									
T2										
2. General ER	0.10 [−0.10, 0.28]	1.00								
3. ER anger	0.06 [−0.14, 0.25]	0.88*** [0.82, 0.92]	1.00							
4. ER sadness	0.08 [−0.11, 0.27]	0.83*** [0.74, 0.89]	0.76*** [0.67, 0.83]	1.00						
5. ER fear	0.09 [−0.10, 0.28]	0.89*** [0.83, 0.93]	0.81*** [0.74, 0.87]	0.74*** [0.64, 0.82]	1.00					
6. ER joy	0.10 [−0.10, 0.29]	0.76*** [0.67, 0.83]	0.55*** [0.41, 0.66]	0.44*** [0.28, 0.57]	0.62*** [0.50, 0.72]					
T3						1.00				
7. INT	0.02 [−0.19, 0.23]	−0.39*** [−0.53, −0.22]	−0.39*** [−0.54, −0.22]	−0.28*** [−0.45, −0.10]	−0.40*** [−0.54, −0.24]	−0.28*** [−0.44, −0.11]	1.00			
8. EXT	−0.11 [−0.31, 0.10]	−0.42*** [−0.57, −0.24]	−0.41*** [−0.58, −0.27]	−0.29*** [−0.46, −0.11]	−0.44*** [−0.58, −0.27]	−0.38*** [−0.52, −0.21]	0.31*** [0.13, 0.47]	1.00		
Covariates										
9. SES	0.12 [−0.07, 0.30]	−0.05 [−0.22, 0.13]	>−0.01 [−0.18, 0.17]	−0.01 [−0.18, 0.16]	0.02 [−0.15, 0.19]	−0.11 [−0.28, 0.07]	0.02 [−0.16, 0.19]	−0.12 [−0.30, 0.06]	1.00	
10. Sex^a^	0.07 [−0.12, 0.26]	0.20* [0.02, 0.36]	0.20* [−0.03, 0.37]	0.12 [−0.05, 0.29]	0.19* [0.01, 0.35]	0.14 [−0.04, 0.30]	−0.08 [−0.26, 0.10]	−0.05 [−0.23, 0.13]	−0.06 [−0.23, 0.11]	

Abbreviations: ER, emotion regulation; EXT, externalizing behavior problems; INT, internalizing behavior problems; SES, socioeconomic status.

^a^ Dummy coded; (0 = boys, 1 = girls).

*p ≤ 0.10, **p ≤ 0.05 ***p ≤ 0.01.

In line with our hypotheses, general ER at 6 years was negatively associated with both INT (*r* = −0.40, *p* < 0.001) and EXT (*r* = −0.42, *p* < 0.001) at 9–10 years. Regulation of all specific emotions (anger, sadness, fear, and joy) were also negatively associated with both INT and EXT (*r* ranging from −0.26 to −0.46; see Table 2). This indicates that higher scores on ER at 6 years were associated with lower scores on INT and EXT at 9–10 years, for both general ER and regulation of specific emotions, when examined independently.

### Path analysis—Longitudinal path models

**FIGURE 1:**
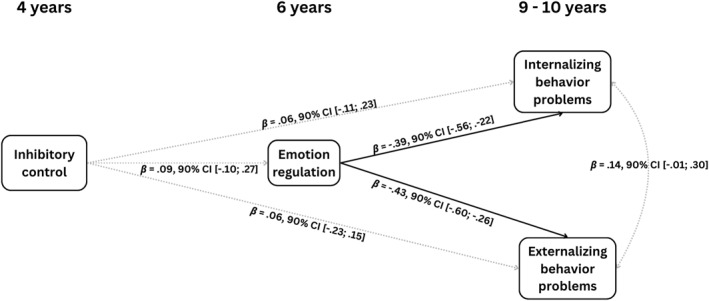
Simplified path diagram depicting results of fitting Model 1 to our data. All path coefficients are standardized. Black, solid lines indicate significant pathways (*α* ≤ 0.10). Gray, dashed lines indicate nonsignificant pathways. Socio‐economic status and child sex were included as covariates in the model, but are excluded from the simplified diagram. See Supporting Information S1: Appendix S3 for further details.

Model 1 illustrates the proposed longitudinal associations between inhibitory control at 4 years, general ER at 6 years, and INT and EXT at 9–10 years, as well as the proposed mediation by general ER. SES and sex were included as covariates in the theoretical model. Results of fitting of Model 1 to our data are shown in Figure 1 (see Supporting Information S1: Appendix S4 for model specification and fit indices). The model was just‐identified with 0 degrees of freedom, meaning that overall model fit indices cannot be evaluated. While this limits the ability to assess global fit, the model specification was theory‐driven, and the obtained path coefficients and confidence intervals provide interpretable information about the associations between variables. After a Bonferroni‐Holm correction, only two coefficients remained statistically significant. No significant covariation was found between INT and EXT at T3 (*b* = 0.14, *p* = 0.13). Based on small effect sizes and high *p*‐values, there was no evidence of the hypothesized longitudinal associations between inhibitory control at T1 and INT (*β* = 0.06, *p* = 0.57) or EXT (*β* = −0.06, *p* = 0.58) at T3, or between inhibitory control and ER at T2 (*β* = 0.09, *p* = 0.43). Conversely, in line with our hypotheses, ER at T2 was significantly negatively associated with INT (*β* = −0.39, *p* < 0.001) and EXT (*β* = −0.43, *p* < 0.001) at T3.

**FIGURE 2:**
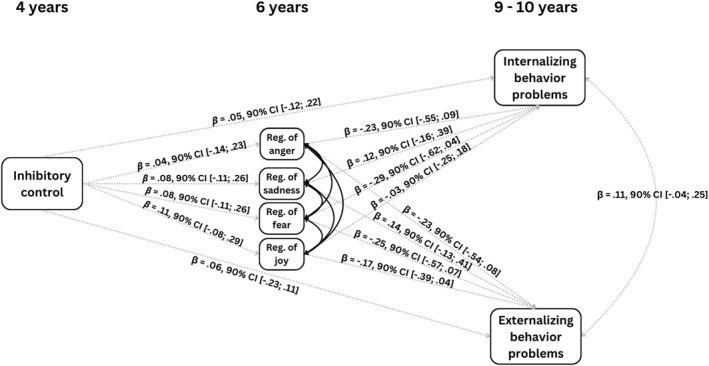
Simplified path diagram depicting results of fitting Model 2 to our data. All path coefficients are standardized. Gray, dashed lines indicate nonsignificant pathways. Black, solid lines indicate significant pathways (here, covariance. See Supporting Information S1: Appendix S4 for covariance coefficients and further details). Socio‐economic status and child sex were included as covariates in the model, but are excluded from the simplified diagram.

Model 2 illustrates the proposed longitudinal associations between inhibitory control at 4 years, regulation of specific emotions at 6 years (anger, fear, sadness, and joy), and INT and EXT at 9–10 years, as well as the proposed mediation by regulation of the specific emotions. Results of fitting Model 2 to our data are shown in Figure 2 (see Supporting Information S1: Appendix S5 for model specification and fit indices). As with Model 1, Model 2 was just‐identified with 0 degrees of freedom. Due to addition of both estimated parameters and variables in Model 2, the number of free parameters equals the number of unique covariances in the data in both models. As previously stated, a just identified model does not preclude meaningful interpretation of path coefficients and confidence intervals. Model 2 contained no statistically significant paths (neither before nor after a Bonferroni‐Holm correction). *β* for theoretically relevant paths ranged from −0.25 to 0.14, with relatively wide confidence intervals, indicating imprecise estimates. The largest, negative effect sizes were observed between regulation of anger and fear to later INT and EXT, while smaller, negative effect sizes were observed between regulation of joy and later INT and EXT, and smaller, positive effect sizes (opposite of expected direction) were observed between regulation of sadness and later INT and EXT (see Figure 2).

## DISCUSSION

Identifying possible malleable predictors of childhood emotional and behavioral problems, as well as underlying developmental processes, is essential for development of successful early intervention. The aim of the current study was to examine specific longitudinal association between inhibitory control in toddlerhood, ER at 6 years, and INT and EXT at 9–10 years, in a typically developing sample. We were interested in the possible mediating role of ER at 6 years in the proposed association between inhibitory control in toddlerhood and INT and EXT at 9–10 years. Additionally, we were interested in examining whether regulation of specific emotions played distinct roles in these associations.

Our results did not support the hypothesized links between toddler inhibitory control and ER at age 6, or INT and EXT at age 9–10. Consequently, we found no evidence for ER as a mediator. However, better general ER at age 6 predicted fewer INT and EXT problems at age 9–10. No statistical significance was observed for paths between regulation of specific emotions and later INT and EXT. While not statistically significant, effect sizes in the expected direction, comparable to those often reported in risk factor studies for psychopathology (see Lynch et al., 2021), were observed between regulation of anger and fear at 6 years, and INT and EXT at 9–10.

### The role of early inhibitory control in later emotion regulation, and INT and EXT

Although inhibitory control in early childhood has previously been associated with later emotional and behavioral problems (Kahle et al., 2018; Lonigan et al., 2017) and ER (Carlson & Wang, 2007; Hughes et al., 2023), we did not find evidence for such associations in the current study. Several factors may explain these results.

Firstly, the associations between inhibitory control and INT and EXT may not have been observed in the current sample due to developmental discontinuity or complexity of associations. Longitudinal associations, especially across several years as in the current study, may be less stable than concurrent associations (Maasalo et al., 2021), and therefore not consistently detected. In addition, several studies suggest a dynamic interplay between these two constructs over time (Maasalo et al., 2021; Quistberg & Mueller, 2020), with no consensus on whether this association is one‐way, or reciprocal (Halse et al., 2024; Thériault‐Couture et al., 2024). Moreover, inhibitory control may have more importance as a protective factor in associations between contextual risk factors and INT and EXT (Hermansen et al., 2022). A general longitudinal association between these variables in a low‐risk sample may therefore be smaller and less detectable.

Secondly, it is possible that the association between inhibitory control and later ER is non‐linear, in that moderate levels of inhibitory control predict better socioemotional outcomes, whereas higher levels of inhibitory control may have negative effects (Carlson & Wang, 2007; Hendry et al., 2022). We did not test for such an association, which, if present, might have weakened the observed associations between the variables by evening out effects. In addition, ER itself is multifaceted, and inhibitory control may support both adaptive and maladaptive strategies. For example, while inhibitory control can be seen as foundational for successful regulation of emotion in general (Bartholomew et al., 2021), it may also be a factor in emotion suppression, a strategy conceptually tied to inhibitory control and associated with INT (Hsieh & Stright, 2012). This complexity highlights the need for distinguishing between ER strategies in future research.

Thirdly, as discussed in Jónsdóttir et al. (2024), the combination of behavioral and parent‐report measures may introduce psychometric challenges (Dang et al., 2020; Hedge et al., 2018). While this approach reduces shared‐method variance, it may have contributed to the lack of associations. Behavioral tasks for young children can be sensitive to measurement error (Byers‐Heinlein et al., 2021; Hendry & Scerif, 2023; Holmboe, 2022). Our one‐time behavioral measurement precluded us from estimating sample‐specific reliability, and low reliability may have reduced power (Byers‐Heinlein et al., 2021). Meanwhile, parent reports in general may be influenced by social desirability, parental bias, or limited insight into the child's internal states. Additionally, the measures may capture different types of performance; behavioral tasks tend to encourage maximal performance (or context dependent performance, see Hendry & Scerif, 2023), while parent‐report may instead indicate typical performance (Dang et al., 2020).

Importantly, reviewing and describing the evidence for an association between inhibitory control and later emotional and behavioral outcomes is hindered by conceptual cluttering and variation of measurement; predictive validity has been found to vary based on method of measurement of inhibitory control (Gagne et al., 2019).

### Emotion regulation as a predictor of INT and EXT

In line with our hypotheses, we found that more successful general ER at 6 years predicted less INT and EXT at 9–10 years. As both INT and EXT were included in the model, specific longitudinal paths to each type of behavior problem could be established. These results indicate that ER in early childhood is a predictor of later variation in both INT and EXT in a community sample. Although our approach was dimensional rather than diagnostic, these findings add to longitudinal evidence linking poor ER to the development of childhood psychopathology (Cavicchioli et al., 2023; Compas et al., 2017), and highlight ER as a plausible, modifiable target for early prevention.

Looking at specific emotions, analysis of our model did not provide any statistically significant paths. The hypothesized mediation and associations between regulation of specific emotions and later behavior problems were therefore not supported. However, the available sample size may have reduced power to detect longitudinal associations. Effect sizes do indicate a possible pattern of associations, which may be detectable in a larger sample. Based on these effect sizes, better regulation of anger and fear, in particular, may be associated with less INT and EXT. Meanwhile, effect sizes between regulation of sadness and joy, and later INT and EXT, were somewhat lower. These observations may relate to the nature of the emotions being regulated; anger and fear in particular may both be characterized as negative emotions, with components of high‐arousal in their expression. Such characteristics of emotional expression, and their (dys)regulation, may play a distinct role in the development of problematic behavior (see e.g. Herman et al., 2018). However, as these associations were not statistically significant, and estimates were imprecise, they should be interpreted with caution, and considered as potential patterns to be examined in future research with larger samples.

### Limitations

Several limitations should be acknowledged. The modest analytic sample, together with attrition across timepoints, reduces statistical power and increases uncertainty of estimates. Selective participation may also have introduced bias, limiting generalizability to clinical or high‐risk populations. Inhibitory control and ER were each measured at a single timepoint, precluding conclusions about developmental change, and the single task measurement of inhibitory control potentially reduces reliability and validity of the construct. Internal consistencies of measurements of both INT and the finer‐grained measures of regulation of specific emotions were modest, which may have reduced the strength of observed associations. Combining behavioral tasks and parent reports reduced shared‐method variance but may also have introduced error due to different response patterns.

Taken together, the value of this study, along with its limitations, point to several important directions for future research.

### Future directions

To optimize the reliability and validity of measurement of inhibitory control, future studies should employ more comprehensive assessments, ideally using repeated measures, and combining multiple measures into a latent construct. Examining specific ER strategies, such as suppression, may clarify both adaptive and maladaptive pathways linking inhibitory control to behavioral and emotional outcomes. Cross‐sectional designs, or studies within shorter developmental windows, could capture the dynamic interdependencies among these processes more precisely, for example by using cross‐lagged models in larger samples. Finally, research in clinically enriched samples will be important for determining whether the observed patterns generalize to populations with heightened regulatory difficulties.

### Conclusions

In a typically developing sample, we examined the longitudinal associations between inhibitory control and both INT and EXT problems from ages 4 to 9–10 years, as well as the potential mediating role of ER at age 6. In contrast to our hypotheses, early inhibitory control did not predict later INT or EXT, and no process of mediation by ER was supported. However, we found evidence of specific, longitudinal associations between ER and subsequent INT and EXT, underscoring the important role that ER plays in child mental health and behavioral adjustment across these symptom dimensions. Our findings reflect the complexity of studying the impact of early inhibitory control on later outcomes, with conceptual and psychometric issues potentially impacting results. Our relatively modest sample size, single‐timepoint assessments, and parent‐report reliabilities, warrant caution in the interpretation of findings, as they may attenuate effects and limit generalizability. Meanwhile, in line with previous empirical evidence and theory, the results highlight general ER as an important target for early intervention, relevant to both INT and EXT trajectories.

## AUTHOR CONTRIBUTIONS

**Lilja K. Jónsdóttir**: Conceptualization; formal analysis; investigation; methodology; writing—original draft; writing—review and editing. **Matilda A. Frick**: Conceptualization; investigation; methodology; writing—review and editing. **Andreas Frick**: Conceptualization; methodology; writing—review and editing. **Emma J. Heeman**: Investigation; methodology; writing—review and editing. **Karin C. Brocki**: Conceptualization; funding acquisition; investigation; methodology; project administration; resources; supervision; writing—review and editing.

## CONFLICT OF INTEREST STATEMENT

The authors declare no conflicts of interest.

## ETHICAL CONSIDERATIONS

The study was approved by the Regional Ethical Board in Uppsala, Sweden, January 20, 2022 (approval number 2021‐05007), and conducted in accordance with the 1964 Declaration of Helsinki. Written informed consent was provided by the children's legal guardians at each timepoint of the study.

## Supporting information

Supporting Information S1

## Data Availability

The data that support the findings of this study are available from the corresponding author upon reasonable request.
