# Supplementary material for: Examining early inhibitory control and emotion regulation as predictors of childhood internalizing and externalizing problems: A longitudinal study
Source: JCPP Adv. 2026 Jan 7;6(3):e70093. doi: 10.1002/jcv2.70093 (PMC13339238; doi:10.1002/jcv2.70093)
Supplement: Supplementary file 1 [file JCV2-6-e70093-s001.docx]

**Examining Early Inhibitory Control and Emotion Regulation as Predictors of Childhood Internalizing and Externalizing Problems:**

**A Longitudinal Study**

**Supporting Information**

**Appendix S1 – Sample**

Initial recruitment of the sample was conducted between August 2013 and June 2014. Parents indicating interest in participating were contacted through mail or phone. In addition to exclusion based on predetermined criteria, further initial attrition was caused by the following; participation declined due to having moved out of the region (*n* = 2), or the project being too time-consuming (*n* = 10).

**Appendix S2 –Analysis code (RMarkdown file knitted to Microsoft Word)**

# Loading necessary packages

library(readr)
library(tidyverse)
library(dplyr)
library(ltm)
library(misty)
library(readxl)

# Data uploading and wrangling

### Uploading dataset

```
BP_data <- read_csv("two_timepoints_with_aux_vars_2024-08-12.csv", show_col_types = FALSE)
BP_data <- subset(BP_data, select = -c(ID) )


BP_data <- BP_data %>%
  rename(age_89y = `age_8-9y`)
BP_data$sex <- ifelse(BP_data$sex == 1, 0,
                      ifelse(BP_data$sex == 2, 1, NA))
```

### Reversing questionnaire items - EQ

```
# Reversing all "self" items on the EQ


library(psych)
keys <- c(1, 1, 1, 1, 1, 1, 1, -1, 1, -1, 1, -1, 1, -1, 1, 1, 1, 1, 1, 1, 1, 1, 1, 1, 1, 1, 1, 1, 1, 1, 1, 1, 1, 1, 1, 1, 1, 1, 1, 1)


BP_data <- reverse.code(keys, BP_data, mini=1, maxi=5)
BP_data
```

### Renaming variables

```
BP_data <- as.data.frame(BP_data)

BP_data <- BP_data %>%
  rename(EQ_sad_self = `EQ_sad_self-`)
BP_data <- BP_data %>%
  rename(EQ_fear_self = `EQ_fear_self-`)
BP_data <- BP_data %>%
  rename(EQ_anger_self = `EQ_anger_self-`)
BP_data <- BP_data %>%
  rename(EQ_joy_self = `EQ_joy_self-`)
```

### Reversing questionnaire items - SDQ

```
# Reversing cp2, pp2, pp3, h4, h5
library(psych)
keys <- c(1, 1, 1, 1, 1, 1, 1, 1, 1, 1, 1, 1, 1, 1, 1, 1, 1, 1, -1, 1, 1, -1, 1, 1, -1, 1, 1, 1, 1, -1, 1, 1, 1, -1, 1, 1, 1, 1, 1, 1)

BP_data <- reverse.code(keys,BP_data,mini=1,maxi=3)
BP_data
```

### Renaming SDQ variables

```
BP_data <- as.data.frame(BP_data)

BP_data <- BP_data %>%
  rename(SDQ_cp2 = `SDQ_cp2-`)
BP_data <- BP_data %>%
  rename(SDQ_pp2 = `SDQ_pp2-`)
BP_data <- BP_data %>%
  rename(SDQ_pp3 = `SDQ_pp3-`)
BP_data <- BP_data %>%
  rename(SDQ_h4 = `SDQ_h4-`)
BP_data <- BP_data %>%
  rename(SDQ_h5 = `SDQ_h5-`)
```

### Cronbach’s alpha for EQ

EQ_data <- data.frame(BP_data$EQ_sad_self, BP_data$EQ_sad_other, BP_data$EQ_fear_self, BP_data$EQ_fear_other, BP_data$EQ_anger_self, BP_data$EQ_anger_other, BP_data$EQ_joy_self, BP_data$EQ_joy_other)
cronbach.alpha(EQ_data, na.rm=TRUE)

### Cronbach’s alpha for SDQ-INT

SDQ_INT <- data.frame(BP_data$SDQ_es, BP_data$SDQ_es2, BP_data$SDQ_es3, BP_data$SDQ_es4, BP_data$SDQ_es5, BP_data$SDQ_pp, BP_data$SDQ_pp2, BP_data$SDQ_pp3, BP_data$SDQ_pp4, BP_data$SDQ_pp5)
cronbach.alpha(SDQ_INT, na.rm=TRUE)

### Cronbach’s alpha for SDQ-EXT

SDQ_EXT <- data.frame(BP_data$SDQ_h, BP_data$SDQ_h2, BP_data$SDQ_h3, BP_data$SDQ_h4, BP_data$SDQ_h5, BP_data$SDQ_cp, BP_data$SDQ_cp2, BP_data$SDQ_cp3, BP_data$SDQ_cp4, BP_data$SDQ_cp5)
cronbach.alpha(SDQ_EXT, na.rm=TRUE)

### Cronbach’s alpha for each emotion

EQ_sad <- data.frame(BP_data$EQ_sad_self, BP_data$EQ_sad_other)
cronbach.alpha(EQ_sad, na.rm=TRUE)

EQ_anger <- data.frame(BP_data$EQ_anger_self, BP_data$EQ_anger_other)
cronbach.alpha(EQ_anger, na.rm=TRUE)

EQ_fear <- data.frame(BP_data$EQ_fear_self, BP_data$EQ_fear_other)
cronbach.alpha(EQ_fear, na.rm=TRUE)

EQ_joy <- data.frame(BP_data$EQ_joy_self, BP_data$EQ_joy_other)
cronbach.alpha(EQ_joy, na.rm=TRUE)

### Create ER variable

BP_data$ER <- rowSums(BP_data[, c("EQ_sad_self", "EQ_sad_other", "EQ_fear_self", "EQ_fear_other", "EQ_anger_self", "EQ_anger_other", "EQ_joy_self", "EQ_joy_other")], na.rm = TRUE)

### Create variables for specific emotions

```
# Anger:
BP_data$ER_anger <- rowMeans(BP_data[, c("EQ_anger_self", "EQ_anger_other")], na.rm = TRUE)

#Fear:
BP_data$ER_fear <- rowMeans(BP_data[, c("EQ_fear_self", "EQ_fear_other")], na.rm = TRUE)

#Sadness:
BP_data$ER_sadness <- rowMeans(BP_data[, c("EQ_sad_self", "EQ_sad_other")], na.rm = TRUE)

#Joy:
BP_data$ER_joy <- rowMeans(BP_data[, c("EQ_joy_self", "EQ_joy_other")], na.rm = TRUE)
```

### Create INT and EXT variables

BP_data$SDQ_INT <- rowSums(BP_data[, c("SDQ_es", "SDQ_es2", "SDQ_es3", "SDQ_es4", "SDQ_es5", "SDQ_pp", "SDQ_pp2", "SDQ_pp3", "SDQ_pp4", "SDQ_pp5")], na.rm = TRUE)

BP_data$SDQ_EXT <- rowSums(BP_data[, c("SDQ_h", "SDQ_h2", "SDQ_h3", "SDQ_h4", "SDQ_h5", "SDQ_cp", "SDQ_cp2", "SDQ_cp3", "SDQ_cp4", "SDQ_cp5")], na.rm = TRUE)

### Removing item specific variables from dataset

BP_data <- subset(BP_data, select = -c(EQ_sad_self, EQ_sad_other, EQ_fear_self, EQ_fear_other, EQ_anger_self, EQ_anger_other, EQ_joy_self, EQ_joy_other, SDQ_es, SDQ_es2, SDQ_es3, SDQ_es4, SDQ_es5, SDQ_pp, SDQ_pp2, SDQ_pp3, SDQ_pp4, SDQ_pp5, SDQ_h, SDQ_h2, SDQ_h3, SDQ_h4, SDQ_h5, SDQ_cp, SDQ_cp2, SDQ_cp3, SDQ_cp4, SDQ_cp5))

### Replacing relevant values with NA

```
# Because I removed NA's from calculating the combined variables, I need to respecify missing values.
library(naniar)


BP_data <- replace_with_na(BP_data, replace = list(ER = 0))

BP_data <- replace_with_na(BP_data, replace = list(SDQ_INT = 0))

BP_data <- replace_with_na(BP_data, replace = list(SDQ_EXT = 0))


BP_data[BP_data == "NaN"] <- NA
```

# Examining dataset - descriptive statistics and distribution

### Descriptive statistics of full dataset

summary(BP_data)

## Main study variables

### DayNight_score (Inhibitory control at 4 years)

### ER (General emotion regulation at 6 years)

psych::describe(BP_data$ER)
hist(BP_data$ER)
class(BP_data$ER)
summary(BP_data$ER)
tabyl(BP_data$ER)
skew(BP_data$ER)/se.skew(BP_data$ER)
Kurtosis(BP_data$ER)/KurtosisSE(BP_data$ER)

### SDQ_INT (Internalizing problems at 8-9 years)

psych::describe(BP_data$SDQ_INT)
hist(BP_data$SDQ_INT)
class(BP_data$SDQ_INT)
summary(BP_data$SDQ_INT)
tabyl(BP_data$SDQ_INT)
skew(BP_data$SDQ_INT)/se.skew(BP_data$SDQ_INT)
Kurtosis(BP_data$SDQ_INT)/KurtosisSE(BP_data$SDQ_INT)

### Box-Cox Transformation of this variable

```
# Fit a simple linear model as a placeholder to use boxcox
model <- lm(SDQ_INT ~ 1, data = BP_data)  # '1' indicates a model with only an intercept

# Apply the Box-Cox transformation to find the best lambda
bc_out <- boxcox(model, lambda = seq(-2, 2, by = 0.1))

# Find the lambda that maximizes the log-likelihood
optimal_lambda <- bc_out$x[which.max(bc_out$y)]
print(paste("Optimal lambda:", optimal_lambda))

# Apply the Box-Cox transformation using the optimal lambda
BP_data$SDQ_INT_transformed <- if (optimal_lambda != 0) {
  (BP_data$SDQ_INT^optimal_lambda - 1) / optimal_lambda
} else {
  log(BP_data$SDQ_INT)
}

par(mfrow = c(1, 2))
hist(BP_data$SDQ_INT, main = "Original Data", xlab = "SDQ_INT")
hist(BP_data$SDQ_INT_transformed, main = "Transformed Data", xlab = "Transformed SDQ_INT")

skew(BP_data$SDQ_INT_transformed)/se.skew(BP_data$SDQ_INT_transformed)
Kurtosis(BP_data$SDQ_INT_transformed)/KurtosisSE(BP_data$SDQ_INT_transformed)
```

### SDQ_EXT (Externalizing problems at 8-9 years)

psych::describe(BP_data$SDQ_EXT)
hist(BP_data$SDQ_EXT)
class(BP_data$SDQ_EXT)
summary(BP_data$SDQ_EXT)
tabyl(BP_data$SDQ_EXT)
skew(BP_data$SDQ_EXT)/se.skew(BP_data$SDQ_EXT)
Kurtosis(BP_data$SDQ_EXT)/KurtosisSE(BP_data$SDQ_EXT)

### Transforming SDQ_EXT

```
# Fit a simple linear model as a placeholder to use boxcox
model <- lm(SDQ_EXT ~ 1, data = BP_data)  # '1' indicates a model with only an intercept

# Apply the Box-Cox transformation to find the best lambda
bc_out <- boxcox(model, lambda = seq(-2, 2, by = 0.1))

# Find the lambda that maximizes the log-likelihood
optimal_lambda <- bc_out$x[which.max(bc_out$y)]
print(paste("Optimal lambda:", optimal_lambda))

# Apply the Box-Cox transformation using the optimal lambda
BP_data$SDQ_EXT_transformed <- if (optimal_lambda != 0) {
  (BP_data$SDQ_EXT^optimal_lambda - 1) / optimal_lambda
} else {
  log(BP_data$SDQ_EXT)
}

par(mfrow = c(1, 2))
hist(BP_data$SDQ_EXT, main = "Original Data", xlab = "SDQ_EXT")
hist(BP_data$SDQ_EXT_transformed, main = "Transformed Data", xlab = "Transformed SDQ_EXT")

skew(BP_data$SDQ_EXT_transformed)/se.skew(BP_data$SDQ_EXT_transformed)
Kurtosis(BP_data$SDQ_EXT_transformed)/KurtosisSE(BP_data$SDQ_EXT_transformed)
```

## Covariates

### SES_index (Socioeconomic status)

psych::describe(BP_data$SES_index)
hist(BP_data$SES_index)
class(BP_data$SES_index)
summary(BP_data$SES_index)
tabyl(BP_data$SES_index)
skew(BP_data$SES_index)/se.skew(BP_data$SES_index)
Kurtosis(BP_data$SES_index)/KurtosisSE(BP_data$SES_index)

### sex (Child biological sex)

tabyl(BP_data$sex)
BP_data$sex <- BP_data$sex - 1

### Correlation matrix using listwise deletion

correlation_matrix <- cor.matrix(BP_data, na.omit = TRUE, method = "spearman")

correlation_matrix

# Outlier detection

### Univariate outlier detection with MAD

### Multivariate outlier detection

# Creating dataset with only relevant variables

```
BP_data_mahalanobis <- BP_data
BP_data_mahalanobis <- subset(BP_data_mahalanobis, select = -c(sex, age_4y, age_6y, age_89y, T1_child_healthprob, T1_parent_ASRS, T2_ABAS_average, T2_child_anxiety_summed, T2_child_ODD_summed, T1_child_SWAN))
BP_data_mahalanobis[] <- lapply(BP_data_mahalanobis, function(x) {
  if(is.numeric(x)) {
    x[is.na(x)] <- mean(x, na.rm = TRUE)
  }
  return(x)
})


means <- colMeans(BP_data_mahalanobis)
cov_matrix <- cov(BP_data_mahalanobis)

# Calculate Mahalanobis distances
distances <- mahalanobis(BP_data_mahalanobis, center = means, cov = cov_matrix)

# Chi-square threshold at 0.01 significance level
threshold <- qchisq(0.99, df = ncol(BP_data_mahalanobis))  # Adjust df to the number of variables

# Find observations with distances exceeding the threshold
outliers <- which(distances > threshold)
print(outliers)

# Create dataset without the outliers

clean_data <- BP_data[-outliers, ]
```

# Missing data

### Exploring missingness in my datasets

```
library(naniar)

# Are there missing values in the dataset?
any_na(BP_data)
# How many?
n_miss(BP_data)
prop_miss(BP_data)
# Which variables are affected?
BP_data %>% is.na() %>% colSums()

# Get number of missings per variable (n and %)
miss_var_summary(BP_data)
miss_var_table(BP_data)
# Get number of missings per participant (n and %)
miss_case_summary(BP_data)
miss_case_table(BP_data)

# Which variables contain the most missing variables?
gg_miss_var(BP_data)
```

### Missing pattern check

library(mice)

PATTERN <- md.pattern(BP_data, rotate.names = T)

PATTERN

### Creating variables indicating missingness for each study variable (value of 1 indicates missingness)

BP_data$DayNight_missing <- as.integer(is.na(BP_data$DayNight_score))

BP_data$ER_missing <- as.integer(is.na(BP_data$ER))

BP_data$INT_missing <- as.integer(is.na(BP_data$SDQ_INT))

BP_data$EXT_missing <- as.integer(is.na(BP_data$SDQ_EXT))

### Testing effects of relevant variables on missingness

```
# What predicts missingness on SDQ_int, SDQ_EXT, DayNight?

## INT

# SES:
t.test(BP_data$SES_index ~ BP_data$INT_missing)

# Inhibitory control:
t.test(BP_data$DayNight_score ~ BP_data$INT_missing)

# Emotion regulation
t.test(BP_data$ER ~ BP_data$INT_missing)

## EXT

# SES:
t.test(BP_data$SES_index ~ BP_data$EXT_missing)

# Inhibitory control:
t.test(BP_data$DayNight_score ~ BP_data$EXT_missing)

# Emotion regulation
t.test(BP_data$ER ~ BP_data$EXT_missing)
```

# Multiple imputation

### Removing missingness variables and original SDQ variables from dataset

BP_data <- subset(BP_data, select = -c(DayNight_missing, ER_missing, INT_missing, EXT_missing, SDQ_INT, SDQ_EXT))

### Scaling variables

BP_data[c('SES_index', 'age_4y', 'age_6y', 'age_89y', 'T1_child_healthprob', 'T1_parent_ASRS', 'T2_ABAS_average', 'T2_child_anxiety_summed', 'T2_child_ODD_summed', 'T1_child_SWAN', 'DayNight_score', 'ER', 'ER_anger', 'ER_sadness', 'ER_fear', 'ER_joy','SDQ_INT_transformed', 'SDQ_EXT_transformed')] <- scale(BP_data[c('SES_index', 'age_4y', 'age_6y', 'age_89y','T1_child_healthprob', 'T1_parent_ASRS', 'T2_ABAS_average', 'T2_child_anxiety_summed', 'T2_child_ODD_summed', 'T1_child_SWAN', 'DayNight_score', 'ER', 'ER_anger', 'ER_sadness', 'ER_fear', 'ER_joy','SDQ_INT_transformed', 'SDQ_EXT_transformed')])

### Creating predictor matrix for multiple imputation using auxiliary variables (missing data is possibly MNAR - do this to reduce bias in imputation)

```
library(mice)

#Creating the matrix
pred_matrix <- make.predictorMatrix(BP_data)

#Specifying the matrix
pred_matrix["DayNight_score", ] <- c(1, 0, 0, 0, 0, 0, 1, 0, 0, 0, 0, 1, 0, 0, 0, 0, 0, 0, 0)  # SES, SWAN-scale score, concurrent child health problems, predict DayNight
pred_matrix["ER", ] <- c(1, 0, 0, 0, 0, 1, 0, 0, 1, 0, 0, 0, 0, 0, 0, 0, 0, 0, 0)  # DayNight score, concurrent ABAS, and SES, predict ER
pred_matrix["ER_anger", ] <- c(1, 0, 0, 0, 0, 1, 0, 0, 1, 0, 0, 0, 0, 0, 0, 0, 0, 0, 0)  # DayNight score, concurrent ABAS, and SES, predict ER_anger
pred_matrix["ER_fear", ] <- c(1, 0, 0, 0, 0, 1, 0, 0, 1, 0, 0, 0, 0, 0, 0, 0, 0, 0, 0)  # DayNight score, concurrent ABAS, and SES, predict ER_fear
pred_matrix["ER_sadness", ] <- c(1, 0, 0, 0, 0, 1, 0, 0, 1, 0, 0, 0, 0, 0, 0, 0, 0, 0, 0)  # DayNight score, concurrent ABAS, and SES, predict ER_sadness
pred_matrix["ER_joy", ] <- c(1, 0, 0, 0, 0, 1, 0, 0, 1, 0, 0, 0, 0, 0, 0, 0, 0, 0, 0)  # DayNight score, concurrent ABAS, and SES, predict ER_joy
pred_matrix["SDQ_INT_transformed", ] <- c(1, 0, 0, 0, 0, 1, 0, 0, 1, 1, 0, 0, 1, 0, 0, 0, 0, 0, 0)  # DayNight score, ER, SES, ABAS at T2, and anxiety symptoms at T2 predict SDQ_INT
pred_matrix["SDQ_EXT_transformed", ] <- c(1, 0, 0, 0, 0, 1, 0, 0, 1, 0, 1, 0, 1, 0, 0, 0, 0, 0, 0)  # DayNight score, ER, SES, ABAS at T2, and ODD symptoms at T2 predict SDQ_EXT
```

### Specifying imputation model

if (!require("mice")) install.packages("mice")

BP_data$sex <- as.factor(BP_data$sex)
BP_data$T1_child_healthprob <- as.factor(BP_data$T1_child_healthprob)

visit_seq <- c("SES_index", "sex", "age_4y", "age_6y", "age_89y",'T1_child_healthprob', 'T1_parent_ASRS', 'T2_ABAS_average', 'T2_child_anxiety_summed', 'T2_child_ODD_summed', "T1_child_SWAN", "DayNight_score", "ER", "ER_anger", "ER_sadness", "ER_fear", "ER_joy","SDQ_INT_transformed", 'SDQ_EXT_transformed')

imputationModel <- mice(BP_data, m=21, seed=500, visitSequence = visit_seq, predictorMatrix = pred_matrix)

### Check imputations

```
summary(imputationModel, action = "long")
plot(imputationModel)
stripplot(imputationModel, pch = 20, cex = 1.2)


# histograms of the imputations vs the observed data.

densityplot(imputationModel, layout = c(1, 1))

# boxplots of the imputations vs the observed data.

bwplot(imputationModel, layout = c(1, 1))
```

# Main analysis

```
# Changing variable classes to be able to calculate correlations:

full.impdata <- complete(imputationModel, 'long', include = TRUE)

full.impdata$sex <- as.numeric(full.impdata$sex)


imputationModel <- as.mids(full.impdata)
```

### Correlations with imputed data

\# Spearman correlations
BP_data$sex <- as.numeric(BP_data$sex)
imp_correlations <- miceadds::micombine.cor(imputationModel, variables=c("DayNight_score","ER", "ER_anger", "ER_sadness", "ER_fear", "ER_joy", "SDQ_INT_transformed", "SDQ_EXT_transformed", "SES_index", "sex"), method = "pearson", partial = c("ER_fear", "ER_joy", "ER_sadness"), conf.level = .90)
imp_correlations

\# Pearson correlations
imp_correlations <- miceadds::micombine.cor(imputationModel, variables=c("DayNight_score","ER", "ER_anger", "ER_sadness", "ER_fear", "ER_joy", "SDQ_INT_transformed", "SDQ_EXT_transformed", "SES_index", "sex"), method = "pearson", conf.level = .90)
imp_correlations

### Checking assumptions for path analysis

```
library(performance)
library(see)

# Model with INT

reg_model1 <- lm(SDQ_INT_transformed ~ DayNight_score + ER, data = BP_data)

par(mfrow = c(2, 2))
plot(reg_model1)
check_model(reg_model1)
check_normality(reg_model1)
check_heteroscedasticity(reg_model1)
check_collinearity(reg_model1)

# Model with EXT

reg_model2 <- lm(SDQ_EXT_transformed ~ DayNight_score + ER, data = BP_data)

par(mfrow = c(2, 2))
plot(reg_model2)
check_model(reg_model2)
check_normality(reg_model2)
check_heteroscedasticity(reg_model2)
check_collinearity(reg_model2)

# Model with EXT

reg_model3 <- lm(SDQ_EXT_transformed ~ DayNight_score + ER_anger + ER_fear + ER_sadness + ER_joy, data = BP_data)

par(mfrow = c(2, 2))
plot(reg_model3)
check_model(reg_model3)
check_normality(reg_model3)
check_heteroscedasticity(reg_model3)
check_collinearity(reg_model3)

reg_model4 <- lm(SDQ_INT_transformed ~ DayNight_score + ER_anger + ER_fear + ER_sadness + ER_joy, data = BP_data)

par(mfrow = c(2, 2))
plot(reg_model4)
check_model(reg_model4)
check_normality(reg_model4)
check_heteroscedasticity(reg_model4)
check_collinearity(reg_model4)


## These checks indicate that assumptions are met.
```

### Specifying path model 1

```
model1 <- '
  # Regression paths

  ER  ~ b1*DayNight_score + e1*sex + f1*SES_index  # ER is predicted by inhibitory control. Sex and SES included as covariates.

  SDQ_INT_transformed ~ c1*DayNight_score + a1*ER + d1*sex + g2*SES_index  # INT is predicted by inhibitory control and ER (mediation). Sex and SES included as covariates.

  SDQ_EXT_transformed ~ c2*DayNight_score + a2*ER + d2*sex + g3*SES_index # EXT is predicted by inhibitory control and ER (mediation). Sex and SES included as covariates.


# Covariance among outcome variables
  SDQ_INT_transformed ~~ SDQ_EXT_transformed  # Covariance between INT and EXT


  # Direct and indirect effects

  # Indirect effects of inhibitory control on INT and EXT through ER
  ind_INT := b1*a1  # indirect effect of inhibitory control on INT through ER
  ind_EXT := b1*a2  # indirect effect of inhibitory control on EXT through ER

  '
```

### Specifying path model 2

```
# Define the model with multiple mediators
model2 <- '
  # Mediator models

  ER_anger ~ b1*DayNight_score + f1*sex + g1*SES_index  # Regulation of anger is predicted by inhibitory control
  ER_sadness ~ b2*DayNight_score + f2*sex + g2*SES_index  # Regulation of sadness is predicted by inhibitory control
  ER_fear ~ b3*DayNight_score + f3*sex + g3*SES_index  # Regulation of fear is predicted by inhibitory control
  ER_joy ~ b4*DayNight_score + f4*sex + g4*SES_index  # Regulation of joy is predicted by inhibitory control

  # Outcome models
  SDQ_INT_transformed ~ c1*DayNight_score + a1*ER_anger + a2*ER_sadness + a3*ER_fear + a4*ER_joy + f5*sex + g5*SES_index  # INT predicted by inhibitory control and specific emotions
  SDQ_EXT_transformed ~ c2*DayNight_score + a5*ER_anger + a6*ER_sadness + a7*ER_fear + a8*ER_joy + d1*sex + g6*SES_index # EXT predicted by inhibitory control and specific emotion

   # Covariance among outcome variables
  SDQ_INT_transformed ~~ SDQ_EXT_transformed  # Covariance between INT and EXT

  # Covariances among mediators
  ER_anger ~~ ER_sadness + ER_fear + ER_joy
  ER_sadness ~~ ER_fear + ER_joy
  ER_fear ~~ ER_joy


  # Indirect effects of inhibitory control on INT through specific emotions
  ind_INT_anger := b1*a1  # through anger
  ind_INT_sadness := b2*a2  # through sadness
  ind_INT_fear := b3*a3  # through fear
  ind_INT_joy := b4*a4  # through joy

  # Indirect effects of inhibitory control on EXT through specific emotions
  ind_EXT_anger := b1*a5  # through anger
  ind_EXT_sadness := b2*a6  # through sadness
  ind_EXT_fear := b3*a7  # through fear
  ind_EXT_joy := b4*a8  # through joy
'
```

### Running path model 1 on imputed data

```
if (!require("lavaan")) install.packages("lavaan")
if (!require("semTools")) install.packages("semTools")

library(semTools)
library(lavaan.mi)


# Running model
fitmodel <- sem.mi(model1, imputationModel)
summary(fitmodel, fit.measures = TRUE, standardized = TRUE,
        rsquare = TRUE, ci = TRUE, level = 0.90)

# if the summary function does not work, use the following:

fitMeasures(fitmodel, c("chisq","df","pvalue","cfi","tli","rmsea","rmsea.ci.lower","rmsea.ci.upper","srmr","aic","bic"),rmsea.ci.level = 0.90)
```

### Running path model 2

```
# Running model 2
fitmodel2 <- sem.mi(model2, imputationModel)
summary(fitmodel2, fit.measures = TRUE, rsquare = TRUE, pool.method = "D2", pool.robust = TRUE, ci = TRUE, level = 0.90, r.squared = TRUE)

# if the summary function does not work, use the following:

fitMeasures(fitmodel2, c("chisq","df","pvalue","cfi","tli","rmsea","rmsea.ci.lower","rmsea.ci.upper","srmr","aic","bic"),rmsea.ci.level = 0.90)
```

### Sensitivity analysis - multivariate outliers

```
  # Fit the path analysis model
fit <- sem(model1, data = clean_data)

# Summarize the results
summary(fit, fit.measures = TRUE, standardized = TRUE, rsquare = TRUE)

# Fit the path analysis model
fit <- sem(model2, data = clean_data)

# Summarize the results
summary(fit, fit.measures = TRUE, standardized = TRUE, rsquare = TRUE)
```

**Appendix S3 – Missing data**

Out of all three timepoints, proportion of missing data on the study variables was highest at T1. Reasons were; no participation at the timepoint (*n* = 7), child failed to understand and follow instructions (*n* = 3), child was unwilling to perform task (*n* = 9), and no reason recorded (*n* = 1). No information describing missingness for the other two timepoints was available, as reasons for not filling out the online questionnaires were not provided.

To reduce bias in our imputation model, a predictor matrix using relevant auxiliary variables measured within the larger project at 4 years; timepoint 1 (T1) or 6 years; timepoint 2 (T2) was created. These auxiliary variables were chosen due to their theoretical association with the given study variables (or possibly with missingness, as in the case of child health problems at T1). This predictor matrix was then used in the multiple imputation.

**Study variables and the predictors used in the imputation model:**

**SES**: all study variables and all auxiliary variables were used as predictors.

**Inhibitory control (T1):** SES, concurrent SWAN-scale score (ADHD symptoms), and concurrent child health problems

**Emotion regulation, and regulation of specific emotions (T2):** SES, score on inhibitory control (T1), concurrent ABAS score (Adaptive Behavior Assessment System – Self-Directions Skills and Social Adaptive Skills subscales averaged).

**Internalizing behavior problems (T3):** SES, score on inhibitory control (T1), score on general emotion regulation (T2), ABAS score (T2), DSM anxiety symptoms (T2)

**Externalizing behavior problems (T3):** SES, score on inhibitory control (T1), score on general emotion regulation (T2), ABAS score (T2), DSM ODD symptoms (T2).

**Correspondence between original data and imputed data**

Density plots comparing distributions of original and imputed values indicate that distributions were well preserved after imputation. This is also true for our measure of Inhibitory Control, which had the largest number of missing values, and therefore the largest risk of instability.

**Appendix S4 –Path model 1: Model specification, fit indices and parameter estimates**

**Model specification – Model 1**

***Emotion regulation*** *~ b1*Inhibitory control + e1*Sex + f1*SES + g1*

(Emotion regulation predicted by inhibitory control. Sex and SES included as covariates).

***Internalizing behavior problems*** *~ c1*Inhibitory control + a1*ER + d1*Sex + g2*SES*

(Internalizing behavior problems predicted by inhibitory control and emotion regulation. Sex and SES included as covariates).

***Externalizing behavior problems*** *~ c2*Inhibitory control + a2*ER + d2*Sex + g3*SES*

(Externalizing behavior problems predicted by inhibitory control and ER. Sex and SES included as covariates).

**Internalizing behavior problems** ~~ **Externalizing behavior problems**

(Estimating covariance between internalizing and externalizing behavior problems)

Indirect effects of inhibitory control on internalizing and externalizing behavior problems through emotion regulation:

**ind_INT** := *b1*a1*

Indirect effect of inhibitory control on INT through ER)

**ind_EXT** := *b1*a2*

Indirect effect of inhibitory control on EXT through ER)

**Fit indices**

**Table S1.** *Fit indices for Model 1.*

| **Fit Index** | **Value** |
| --- | --- |
| Comparative Fit Index (CFI) | 1.000 |
| *df* | 0.000 |
| Standardized Root Mean Square Residual, (SRMR) \| \| \| | 0.061 |
| Chi-Square (X²) | 0.000 |
| *p*-value, | 1.000 |
| Root Mean Square Error of Approximation, (RMSEA) \| | 0.000 |
| Akaike Information Criterion (AIC) \| | 784.403 |
| Bayesian Information Criterion (BIC) \| | 822.552 |

**Parameter estimates**

**Table S2**. *Parameter estimates for Model 1 – direct and indirect effects*

|  | ***β*** | **SE** | ***p*** | **Lower 90% CI** | **Upper 90% CI** |
| --- | --- | --- | --- | --- | --- |
| Inhibitory control 🡪 ER | 0.089 | 0.111 | 0.426 | -0.095 | 0.272 |
| Sex 🡪 ER | 0.375 | 0.221 | 0.090 | -0.011 | 0.738 |
| SES 🡪 ER | -0.046 | 0.112 | 0.679 | -0.230 | 0.137 |
| Inhibitory control 🡪 INT | 0.060 | 0.105 | 0.570 | -0.114 | 0.233 |
| ER 🡪 INT | -0.390 | 0.106 | 0.000 | -0.564 | -0.216 |
| Sex 🡪 INT | -0.017 | 0.211 | 0.937 | -0.363 | 0.330 |
| SES 🡪 INT | -0.008 | 0.105 | 0.943 | -0.179 | 0.164 |
| Inhibitory control 🡪 EXT | -0.057 | 0.103 | 0.583 | -0.227 | 0.114 |
| ER 🡪 EXT | -0.427 | 0.104 | 0.000 | -0.599 | -0.256 |
| Sex 🡪 EXT | 0.051 | 0.207 | 0.806 | -0.290 | 0.391 |
| SES 🡪 EXT | -0.136 | 0.103 | 0.187 | -0.305 | 0.034 |
| Covariance:  INT and EXT | 0.143 | 0.093 | 0.125 | -0.010 | 0.296 |
| Variance: ER | 0.940 | 0.150 | 0.000 | 0.693 | 1.187 |
| Variance: INT | 0.822 | 0.132 | 0.000 | 0.606 | 1.039 |
| Variance: EXT | 0.794 | 0.127 | 0.000 | 0.585 | 1.003 |
| **Indirect effect:**  Inhibitory control 🡪 ER 🡪 INT | -0.035 | 0.048 | 0.472 | -0.114 | 0.045 |
| **Indirect effect:** Inhibitory control 🡪 ER 🡪 EXT | -0.037 | 0.049 | 0.449 | -0.118 | 0.044 |

**Appendix S5 –Path model 2: Model specification, fit indices and parameter estimates**

**Model specification – Model 2**

***ER anger*** *~ b1*Inhibitory control + f1*Sex + g1*SES*
Emotion regulation of anger is predicted by inhibitory control, with sex and SES as covariates.

***ER sadness*** *~ b2*Inhibitory control + f2*Sex + g2*SES*
Emotion regulation of sadness is predicted by inhibitory control, with sex and SES as covariates.

***ER fear*** *~ b3*Inhibitory control + f3*Sex + g3*SES*
Emotion regulation of fear is predicted by inhibitory control, with sex and SES as covariates.

***ER joy*** *~ b4*Inhibitory control + f4*Sex + g4*SES*
Emotion regulation of joy is predicted by inhibitory control, with sex and SES as covariates.

***Internalizing behavior problems*** *~ c1*Inhibitory control + a1*ER anger + a2*ER sadness + a3*ER fear + a4*ER joy + f5*Sex + g5*SES*
Internalizing behavior problems are predicted by inhibitory control and specific emotions (anger, sadness, fear, and joy), with sex and SES as covariates.

***Externalizing behavior problems*** *~ c2*Inhibitory control + a5*ER anger + a6*ER sadness + a7*ER fear + a8*ER joy + d1*Sex + g6*SES*
Externalizing behavior problems are predicted by inhibitory control and specific emotions (anger, sadness, fear, and joy), with sex and SES as covariates.

***Internalizing behavior problems*** *~~* ***Externalizing behavior problems***
Estimating covariance between internalizing and externalizing behavior problems.

Estimating covariance among emotion regulation variables:

***ER anger*** *~~* ***ER sadness*** *+* ***ER fear*** *+* ***ER joy***

***ER sadness*** *~~* ***ER fear*** *+* ***ER joy***

***ER fear*** *~~* ***ER joy***

**ind_INT_anger** := *b1*a1*
Indirect effect of inhibitory control on internalizing behavior problems through emotion regulation of anger.

**ind_INT_sadness** := *b2*a2*
Indirect effect of inhibitory control on internalizing behavior problems through emotion regulation of sadness.

**ind_INT_fear** := *b3*a3*
Indirect effect of inhibitory control on internalizing behavior problems through emotion regulation of fear.

**ind_INT_joy** := *b4*a4*
Indirect effect of inhibitory control on internalizing behavior problems through emotion regulation of joy.

**ind_EXT_anger** := *b1*a5*
Indirect effect of inhibitory control on externalizing behavior problems through emotion regulation of anger.

**ind_EXT_sadness** := *b2*a6*
Indirect effect of inhibitory control on externalizing behavior problems through emotion regulation of sadness.

**ind_EXT_fear** := *b3*a7*
Indirect effect of inhibitory control on externalizing behavior problems through emotion regulation of fear.

**ind_EXT_joy** := *b4*a8*
Indirect effect of inhibitory control on externalizing behavior problems through emotion regulation of joy.

**Fit indices**

**Table S3.** *Fit indices for Model 2*

| **Fit Index** | **Value** |
| --- | --- |
| Comparative Fit Index (CFI) | 1.000 |
| *df* | 0.000 |
| Standardized Root Mean Square Residual (SRMR) | 0.035 |
| Chi-Square (X²) | 0.000 |
| P-value | 1.000 |
| Root Mean Square Error of Approximation (RMSEA) | 0.000 |
| Akaike Information Criterion (AIC) | 1367.800 |
| Bayesian Information Criterion (BIC) | 1466.989 |

**Parameter estimates**

**Table S4**. *Parameter estimates for Model 2 – direct and indirect effects.*

|  | ***β*** | **SE** | ***p*** | **Lower 90% CI** | **Upper 90% CI** |
| --- | --- | --- | --- | --- | --- |
| Inhibitory control 🡪 ER anger | 0.042 | 0.112 | 0.708 | -0.142 | 0.226 |
| Sex 🡪 ER anger | 0.404 | 0.222 | 0.068 | 0.039 | 0.768 |
| SES 🡪 ER anger | 0.000 | 0.112 | 0.998 | -0.184 | 0.184 |
| Inhibitory control 🡪 ER sadness | 0.075 | 0.112 | 0.507 | -0.111 | 0.260 |
| Sex 🡪 ER sadness | 0.232 | 0.223 | 0.299 | -0.135 | 0.598 |
| SES 🡪 ER sadness | -0.014 | 0.113 | 0.900 | -0.199 | 0.171 |
| Inhibitory control 🡪 ER fear | 0.078 | 0.112 | 0.484 | -0.106 | 0.262 |
| Sex 🡪 ER fear | 0.362 | 0.222 | 0.102 | -0.002 | 0.727 |
| SES 🡪 ER fear | 0.023 | 0.112 | 0.841 | -0.162 | 0.207 |
| Inhibitory control 🡪 ER joy | 0.106 | 0.112 | 0.344 | -0.078 | 0.290 |
| Sex 🡪 ER joy | 0.244 | 0.221 | 0.270 | -0.120 | 0.608 |
| SES 🡪 ER joy | -0.116 | 0.112 | 0.298 | -0.336 | 0.105 |
| Inhibitory control 🡪 INT | 0.052 | 0.104 | 0.615 | -0.119 | 0.223 |
| ER anger 🡪 INT | -0.230 | 0.194 | 0.235 | -0.549 | 0.089 |
| ER sadness 🡪 INT | 0.118 | 0.167 | 0.480 | -0.157 | 0.349 |
| ER fear 🡪 INT | -0.286 | 0.200 | 0.152 | -0.615 | 0.043 |
| ER joy 🡪 INT | -0.034 | 0.133 | 0.799 | -0.252 | 0.184 |
| Sex 🡪 INT | 0.016 | 0.208 | 0.940 | -0.326 | 0.358 |
| SES 🡪 INT | 0.014 | 0.104 | 0.891 | -0.157 | 0.185 |
| Inhibitory control 🡪 EXT | -0.060 | 0.100 | 0.552 | -0.226 | 0.106 |
| ER anger 🡪 EXT | -0.230 | 0.187 | 0.220 | -0.539 | 0.079 |
| ER sadness 🡪 EXT | 0.139 | 0.162 | 0.392 | -0.128 | 0.406 |
| ER fear 🡪 EXT | -0.250 | 0.194 | 0.197 | -0.569 | 0.069 |
| ER joy 🡪 EXT | -0.174 | 0.128 | 0.176 | -0.385 | 0.037 |
| Sex 🡪 EXT | 0.087 | 0.202 | 0.665 | -0.244 | 0.418 |
| SES 🡪 EXT | -0.129 | 0.101 | 0.200 | -0.295 | 0.037 |
| Covariance:  INT and EXT | 0.109 | 0.087 | 0.211 | -0.035 | 0.253 |
| Covariance:  ER anger ~~ ER sadness | 0.723 | 0.135 | 0.000 | 0.501 | 0.946 |
| Covariance: ER anger ~~ ER fear | 0.765 | 0.138 | 0.000 | 0.539 | 0.992 |
| Covariance: ER anger ~~ ER joy | 0.509 | 0.121 | 0.000 | 0.309 | 0.709 |
| Covariance:  ER sadness ~~ ER fear | 0.701 | 0.134 | 0.000 | 0.481 | 0.921 |
| Covariance:  ER sadness ~~ ER joy | 0.407 | 0.117 | 0.001 | 0.215 | 0.600 |
| Covariance:  ER fear ~~ ER joy | 0.584 | 0.126 | 0.000 | 0.377 | 0.791 |
| Variance: ER anger | 0.948 | 0.151 | 0.000 | 0.699 | 1.197 |
| Variance: ER sadness | 0.961 | 0.153 | 0.000 | 0.708 | 1.213 |
| Variance: ER fear | 0.949 | 0.152 | 0.000 | 0.700 | 1.198 |
| Variance: ER joy | 0.946 | 0.151 | 0.000 | 0.697 | 1.194 |
| Variance: INT | 0.792 | 0.127 | 0.000 | 0.584 | 1.006 |
| Variance: EXT | 0.741 | 0.119 | 0.000 | 0.546 | 0.936 |
| **Indirect effect:**  Inhibitory control 🡪 ER anger 🡪 INT | -0.009 | 0.035 | 0.804 | -0.078 | 0.050 |
| **Indirect effect**:  Inhibitory control 🡪 ER sadness 🡪 INT | 0.008 | 0.010 | 0.415 | -0.051 | 0.072 |
| **Indirect effect:**  Inhibitory control 🡪 ER fear 🡪 INT | -0.023 | 0.042 | 0.584 | -0.111 | 0.068 |
| **Indirect effect:**  Inhibitory control 🡪 ER joy 🡪 INT | -0.004 | 0.024 | 0.874 | -0.043 | 0.035 |
| **Indirect effect:**  Inhibitory control 🡪 ER anger 🡪EXT | -0.009 | 0.030 | 0.772 | -0.090 | 0.060 |
| **Indirect effect:**  Inhibitory control 🡪 ER sadness 🡪 EXT | 0.011 | 0.010 | 0.275 | -0.030 | 0.057 |
| **Indirect effect:**  Inhibitory control 🡪 ER fear 🡪 EXT | -0.019 | 0.047 | 0.695 | -0.057 | 0.035 |
| **Indirect effect:**  Inhibitory control 🡪 ER joy 🡪 EXT | -0.018 | 0.026 | 0.500 | -0.061 | 0.025 |
